# Identification of hub gene and lncRNA signature related to entotic cell death in cutaneous melanoma for prognostic and immune prediction

**DOI:** 10.1097/MD.0000000000035881

**Published:** 2023-11-10

**Authors:** Chen Zhang, Chenyang Shen

**Affiliations:** a Department of Emergency Surgery, Linping Campus, Second Affiliated Hospital, Zhejiang University School of Medicine, Hangzhou, China; b Department of Infectious Disease, Linping Campus, Second Affiliated Hospital, Zhejiang University School of Medicine, Hangzhou, China.

**Keywords:** cutaneous melanoma, entotic cell death, gene, immune microenvironment, lncRNA

## Abstract

Entotic cell death (ECD), a cell death program observed in cancer cell competition, predominantly occurs in an autophagy protein-dependent, non-apoptotic manner. However, the relationship between cutaneous melanoma (CM) and ECD-associated genes and lncRNAs has remained unclear. This study aimed to elucidate the role and mechanism of ECD-associated genes in CM. To achieve this, 4 mechanism learning algorithms and integrated bioinformatic analyses were employed to identify the core ECD-associated genes and lncRNAs. Subsequently, 2 risk signatures based on ECD-associated genes and hub lncRNAs were constructed for CM patients. As a result, we observed significant differential expression of ECD-associated genes in CM, indicating their potential as valuable predictors for CM patients. Moreover, *RHOA* was identified as a core ECD-associated gene in CM, and its expression was found to be associated with patients’ survival and immune infiltration, suggesting its relevance as a potential therapeutic target. Additionally, this study provided clarification on hub ECD-associated lncRNAs in CM, offering insights into their roles in the disease. Through bioinformatic analyses, we identified 2 risk signatures based on the expression of ECD-associated genes and hub ECD-associated lncRNAs, respectively. Both risk signatures were strongly linked to the prognosis and cancer growth of CM, underscoring their potential as valuable prognostic indicators. Furthermore, mechanistic analyses suggested a significant association between the risk signature and the immune microenvironment in CM, highlighting potential immune-related implications in disease progression. In conclusion, we propose that ECD-associated genes and lncRNAs hold promise as potential targets in CM. Moreover, our findings revealed a significant correlation between ECD and the immune microenvironment, providing crucial insights for guiding individualized treatment strategies in CM.

## 1. Introduction

Cutaneous melanoma (CM), a malignant tumor,^[1]^ ranks fifth among prevalent cancers in male patients and sixth in female patients, according to the 2020 cancer statistics.^[2]^ CM is considered curable by extended surgical resection at the early stage, with a 5-year survival probability of approximately 40% to 50%.^[3]^ Unfortunately, the majority of CM patients are diagnosed with advanced metastatic CM, leading to a significant reduction in their 5-year survival prognosis, which stands at 19%.^[4,5]^ Personalized therapy and prognostic prediction for CM necessitate the identification and utilization of specific biomarkers. However, the current list of biomarkers identified with clinical significance for CM is insufficient.^[6]^ Therefore, the identification and screening of novel biomarkers, along with the construction of prognostic signatures capable of accurately predicting a patient’s condition, become urgent priorities.

Entotic cell death (ECD) is a novel cell death mechanism characterized by the formation of “cell-in-cell” structures in cancer. This process is primarily observed in sibling cells, where cancer cells identify and adhere to neighboring cells through the epithelial adherens junction, which consists of E-cadherin and the cytoskeleton linker protein α-catenin.^[7,8]^ Once cell-cell adhesion is formed, the ingested cell zealously invades into the other cancer cell through increased RhoA-GTPase and ROCK-dependent actomyosin contraction.^[9,10]^ In contrast to traditional cell death mechanisms like anoikis and apoptosis, ECD degrades the internalized cells through lysosome fusion, without triggering nuclear fragmentation, caspase-3/-1/-11 cleavage, or altering rapamycin activity.^[11]^ While some engulfed cells can escape from their hosts, most undergo primarily a death process, suggesting that ECD might serve as a suppression mechanism of cancer cells.^[11]^ However, the precise mechanisms responsible for the potential role of ECD in tumor suppression remain unclear. Additionally, the association between ECD and CM has yet to be explored.

Recent progress in bioinformatic analyses has enabled researchers to identify numerous biomarkers that are specific to various diseases. Despite of that, Recent progress in bioinformatic analyses has enabled researchers to identify numerous disease-specific biomarkers. However, there is a lack of identifiable genes associated with ECD and the prognosis/progression of CM. Thus, we conducted a screening of hub ECD-associated genes and lncRNAs significantly associated with CM patient prognosis. Subsequently, based on the expressions of ECD-associated genes and lncRNAs, we constructed 2 risk signatures for CM patients. The clinical significance and prognostic role of these risk signatures were also evaluated. Furthermore, we observed close connections between ECD-associated genes and the risk signature with the immune microenvironment. In summary, this study is the first to identify the role of ECD-associated genes and lncRNAs in CM, presenting a promising target for predicting prognosis and clinical outcomes in CM patients.

## 2. Materials and methods

### 2.1. Acquisition of raw data

RNA sequencing datasets of 471 CM patients with clinical information were collected from The Cancer Genome Atlas (TCGA, https://portal.gdc.cancer.gov/) database. Additionally, to address the lack of control samples (only 1 normal skin) in the TCGA database, 812 normal skin samples were obtained from the Genotype-Tissue Expression (http://commonfund.nih.gov/GTEx/) database. To facilitate comparative analysis of gene expression data obtained from different platforms, the “limma” R package was employed to normalize the data and convert it into a single matrix. Subsequently, the “sva” R package was used to remove batch effects. Based on prior research,^[12]^ ECD-associated genes (*AMPK, ATG5, ATG7, BECN1, CDC42, CDH1, CTNNA1, CYBB, MYH14, PI3KC3, RHOA, RNF146, ROCK, RUBCN*, and *UVRAG*) were identified through screening. However, given our observation that *AMPK, PI3KC3*, and *ROCK* were not expressed in CM tissues, they were excluded from this study, and the remaining 12 ECD-associated genes were subjected to further analysis. For external validation, additional CM samples were collected from the GEO-GSE65904 cohort. CNV data of CM patients were also obtained from the TCGA database. The search of all public databases in this study was conducted in compliance with the relevant guidelines. As it did not involve human or animal subjects, ethical approval was not required from the Ethics Committee of the First People’s Hospital of Linping District.

### 2.2. Machine learning model construction

ECD-associated genes were subjected to construct 4 different machine learning models, including Generalized Linear Models, Random Forest, Support Vector Machine, and Extreme Gradient Boosting, using R packets “xgboost,” “random-Forest,” “dalex,” and “caret,” respectively. These 4 machine learning tasks were conducted using the “kernlab” R package. To assess the accuracy of these mechanism models, residual analysis was applied to the samples, and the reverse cumulative distribution of residuals was plotted to evaluate the models. The receiver operating characteristic (ROC) curves of the 4 machine learning models were plotted using the “pROC” R package. To identify key genes for CM patients, each ECD-associated gene in the models was assigned an importance score. Based on the results of the 4 machine learning models, the hub ECD-associated gene in CM was identified, and KM survival analysis, along with ROC analysis, was performed for the core gene.

### 2.3. Functional enrichment analyses

Firstly, the Database for Annotation, Visualization, and Integrated Discovery (version 6.8) was utilized for Gene Ontology (GO) analysis to determine the biological functions of ECD-associated genes. GO enrichment analyses were conducted for cellular components, molecular function, and biological processes. Thereafter, GSEA and GSVA analyses were performed to investigate the potential mechanism of the hub-identified ECD-associated gene *RHOA*. GSEA was conducted on the gene expression matrix using the “c2.cp.kegg.symbols.gmt” sets to detect Kyoto Encyclopedia of Genes and Genomes pathways. According to the GSVA score matrix, gene-level changes were transformed into pathway-level ones using the “GSVA” R package, ultimately enabling the evaluation of potential biological functions. Statistical significance was defined as false discovery rate and *P* values < .05.

### 2.4. Screening for overlapping hub ECD-associated lncRNAs

The identification of hub lncRNAs involved performing Pearson correlation analysis (|R2| > 0.4, *P* < .05) to assess the connections between CM and ECD-associated lncRNAs, followed by univariate Cox regression analysis, differential expression analysis, and WGCNA. The cutoff criterion of *P* < .01 was utilized in the univariate Cox regression analysis. The R package “limma” was utilized to conduct differential expression analysis for ECD-associated lncRNAs in CM, employing criteria of |log fold change| > 1 and *P* < .05. With the assistance of the R package “WGCNA,” we established the co-expression network linking ECD-associated lncRNAs with sample modules. Based on the scale-free topological criterion (>0.93) and a minimum power value, the best soft threshold was determined to be five. Later, the weighted adjacency matrix was used to generate a topological overlap matrix, and hierarchical clustering was applied to construct dendrograms based on the topological overlap matrix. To control the number of modules generated, the main parameters were defined with a minModuleSize of 10. In conclusion, ECD-associated lncRNAs displaying notable interconnections were assigned to various patterns. The calculation of gene significance and module eigengenes was carried out for all modules. The “VennDiagram” package was utilized to visualize the overlap of lncRNAs among WGCNA, differential expression, and univariate Cox regression analyses, and these shared lncRNAs were designated as hub ECD-associated lncRNAs for further investigation. The “survival” R package was utilized for KM survival analysis, and the “limma” R package was employed for differential expression analysis. The predictive accuracy of hub lncRNAs were verified using the R package “pROC.”

### 2.5. Construction of the gene and lncRNA signatures

Gene and lncRNA risk signatures were generated based on the expression of 11 ECD-associated genes and 3 identified hub ECD-associated lncRNAs, respectively. These selected lncRNAs and genes were applied into a Lasso penalized Cox regression analysis, and the risk score of signatures were constructed using the following formula:

risk score = Σexpi * βi

where expi represents the relative expression of ECD-associated genes or lncRNAs i, and β is the regression coefficient. Then, based on the median value of the constructed risk score, CM patients were segregated into low- and high-risk subgroups.

The “ggplot2” and “Rtsne” packages were employed, and PCA and t-SNE were conducted to investigate the distribution of risk subgroups. Then, Cox regression and KM survival analyses were employed to assess and compare the prognostic ability. Next, the “timeROC” and “cliROC” packages were applied to calculate the accuracy of this risk signatures for prediction. We utilized the “rms” package to construct a nomogram that utilized the risk scores of the gene signature for predicting CM patients’ outcomes, and the accuracy and discrimination were subsequently evaluated through decision curve analysis.

### 2.6. Immune correlation analysis

Firstly, CIBERSORT analysis was conducted to assess the relationship between the expression of ECD-associated genes and immune cell infiltration. Thereafter, single-sample GSEA was performed to explore the discernible differences in immune cell infiltration and immune functions among CM risk subgroups. The relationship between the scores of immune-associated factors (including stromal and immune scores) and the risk signature was evaluated by Spearman correlation analysis. By referring to the potential immune checkpoints identified in a previous study, we ascertained the link between the risk signature and immune-associated genes.^[13]^ Next, the connections of the risk signature to *PD-L2* and *PD-L1* were clarified. By employing Spearman correlation analyses, we assessed the associations between m6A-related genes, tumor stemness, and risk score.

## 3. Results

### 3.1. Expression and mechanism of ECD-associated genes in CM

**Figure 1.**
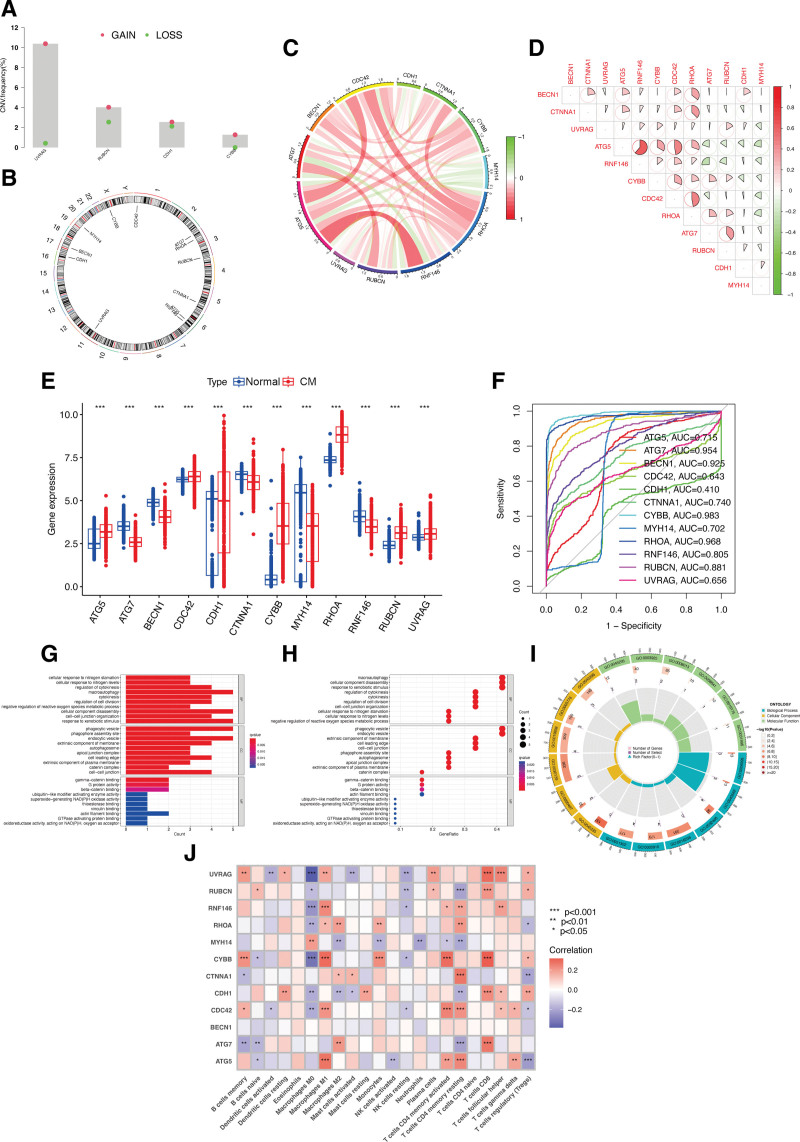
The role of ECD-associated genes in CM. (A) The CNV variation frequency of ECD-associated genes. (B) The location of CNV alteration of ECD-associated genes on 23 chromosomes. (C and D) The correlation between 12 ECD-associated genes. (E) The expression difference of ECD-associated genes between normal and CM samples. (F) ROC curve of ECD-associated genes for CM. The GO enrichment terms of ECD-associated genes in CC, BP, and MF are shown by a bar plot (G), bubble chart (H), and circle plot (I). (J) Correlation heatmap depicting correlations between infiltrated immune cells and ECD-associated genes in CM. BP = biological processes, CC = cellular components, CM = cutaneous melanoma, CNVs = copy number variations, ECD = entotic cell death, GO = gene ontology, MF = molecular function, ROC = receiver operating characteristic.

Investigation of somatic copy number variations (CNVs) in ECD-associated genes in CM revealed a higher prevalence of CNVs in the genes *UVRAG, RUBCN, CDH1, and CYBB. Specifically, both UVRAG, RUBCN, CDH1*, and *CYBB* exhibited increased CNV changes (Fig. 1A). The alterations of CNVs across the respective chromosomes of ECD-associated genes are depicted in Figure 1B. Interestingly, network visualization of the correlation between ECD-associated genes showed that all genes exhibited significant associations with each other (Fig. 1C and D). The expression levels of ECD-associated genes *ATG5, CDC42, CYBB, RHOA, RUBCN*, and *UVRA*G *were significantly evaluated in CM samples compared to normal skin tissues. However, the expression of genes ATG7, BECN1, CDH1, CTNNA1, MYH14*, and *RNF146* were significantly decreased in CM (Fig. 1E). To further confirm the predictive accuracy of ECD-associated genes in predicting the occurrence of CM, it was discovered that only *CDH1, CDC42*, and *UVRAG* showed moderate ROC value, while the other genes specifically predicted CM (ROC > 0.7; Fig. 1F).

Functional analyses were conducted based on the intersection of ECD-associated genes. Regarding biological processes, the genes were mostly enriched in cellular response to nitrogen starvation, regulation of cytokinesis, and macroautophagy. In terms of cellular components, the genes were linked to phagocytic vesicle, phagophore assembly site, and endocytic vesicle. As for molecular function, the genes were mainly involved in gamma-catenin binding, G protein activity, and beta-catenin binding (Fig. 1G). Figure 1H and I presented a bubble chart and Circos diagram of the GO enrichment analysis, respectively. To investigate the relationship of ECD-associated genes with immune infiltration in CM, Figure 1J indicated that 12 ECD-associated genes exhibited a significant correlation with the proportions of immune cells, thereby providing evidence for the potential of ECD-associated genes as prognostic targets for CM immunotherapy.

### 3.2. Risk signature of ECD-associated genes for CM patients

**Figure 2.**
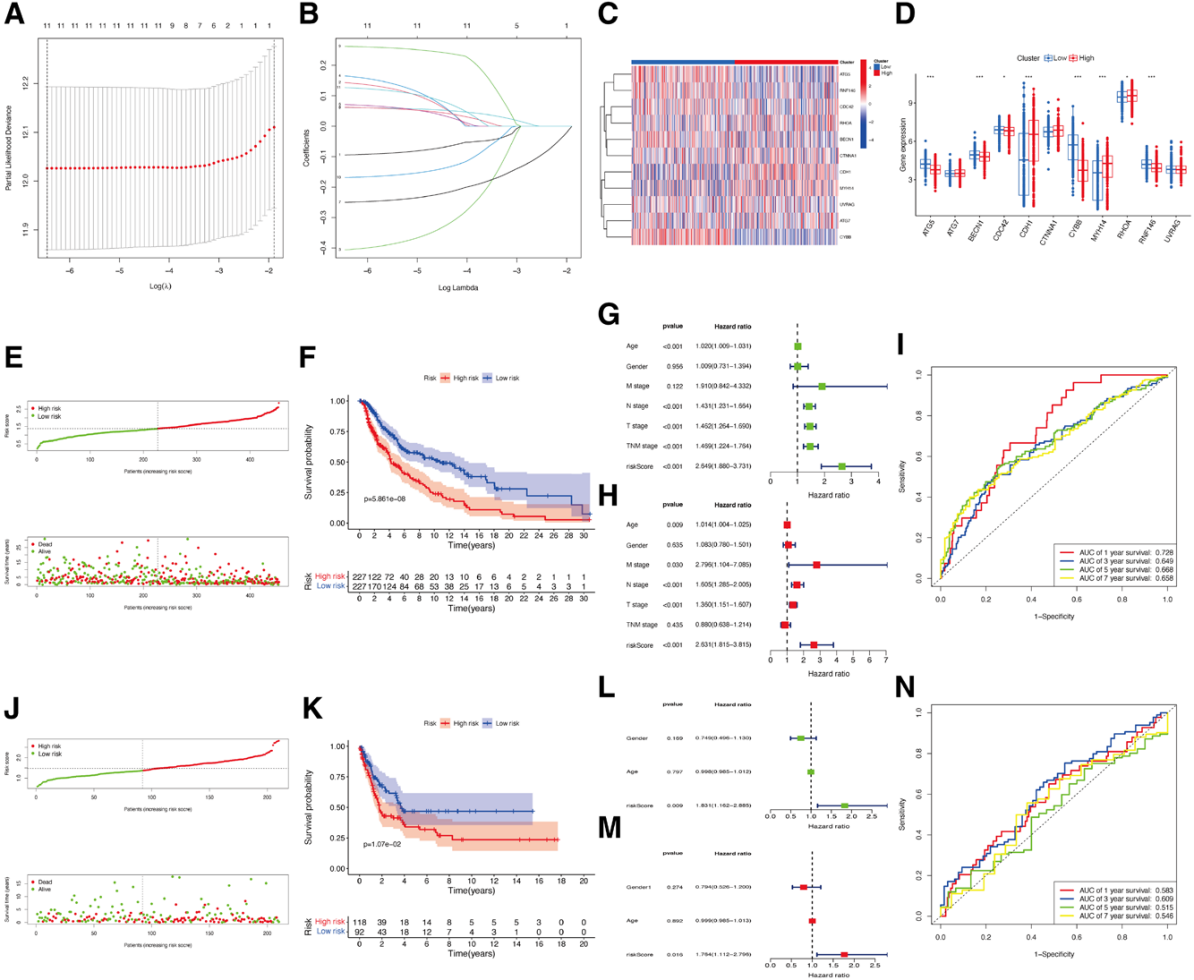
Construction of ECD-associated genes risk signature. (A and B) LASSO analysis to determine factors and construct the model. (C and D) The differential expression of ECD-associated genes in the risk subgroups. (E–H) The risk score distributions and survival status in the TCGA-CM set. (J–M) The risk score distributions and survival status in the GSE65904 set. CM = cutaneous melanoma, ECD = entotic cell death, TCGA = The Cancer Genome Atlas.

For the construction of the risk signature model, the LASSO analysis was applied for ECD-associated genes and 11 hub genes, namely *ATG5, ATG7, BECN1, CDC42, CDH1, CTNNA1, CYBB, MYH14, RHOA, RNF146*, and *UVRAG* (Fig. 2A and B). During the investigation of the connection between ECD-associated genes and risk subgroups, it was observed that genes *CDH1, MYH14*, and *RHOA* were significant higher expressed in high-risk subgroup, whereas *ATG5, BECN1, CDC42, CYBB*, and *RNF146* was lower expressed in high-risk subgroup with statistical significance (Fig. 2C and D). According to the calculated median value of risk scores, 2 subgroups were classified as high- or low-risk score for the patients from the cohorts of TCGA-CM (Fig. 2E) and GSE65904 (Fig. 2J). The KM survival analysis indicated that in both TCGA and GEO cohort, the CM patients OS is negatively correlated with their risk scores (*P* < .05; Fig. 2F and K). Additionally, our analysis also showed that, while compared with other clinical features in CM patients, the risk score was not only served as an independent prognostic factor (Fig. 2H and M), but also significantly associated with prognosis (Fig. 2G and L). Meanwhile, in the cohort of TCGA-CM, the AUC values for the moderate predictive accuracy contained in our risk signature at 1-year, 3-year, 5-year, and 7-year follow-up are 0.728, 0.649, 0.668, and 0.658 respectively (Fig. 2I). The AUC in the cohort for validation at 1-year, 3-year, 5-year, and 7-year follow-up are 0.583, 0.609, 0.515, and 0.546 respectively (Fig. 2N).

**Figure 3.**
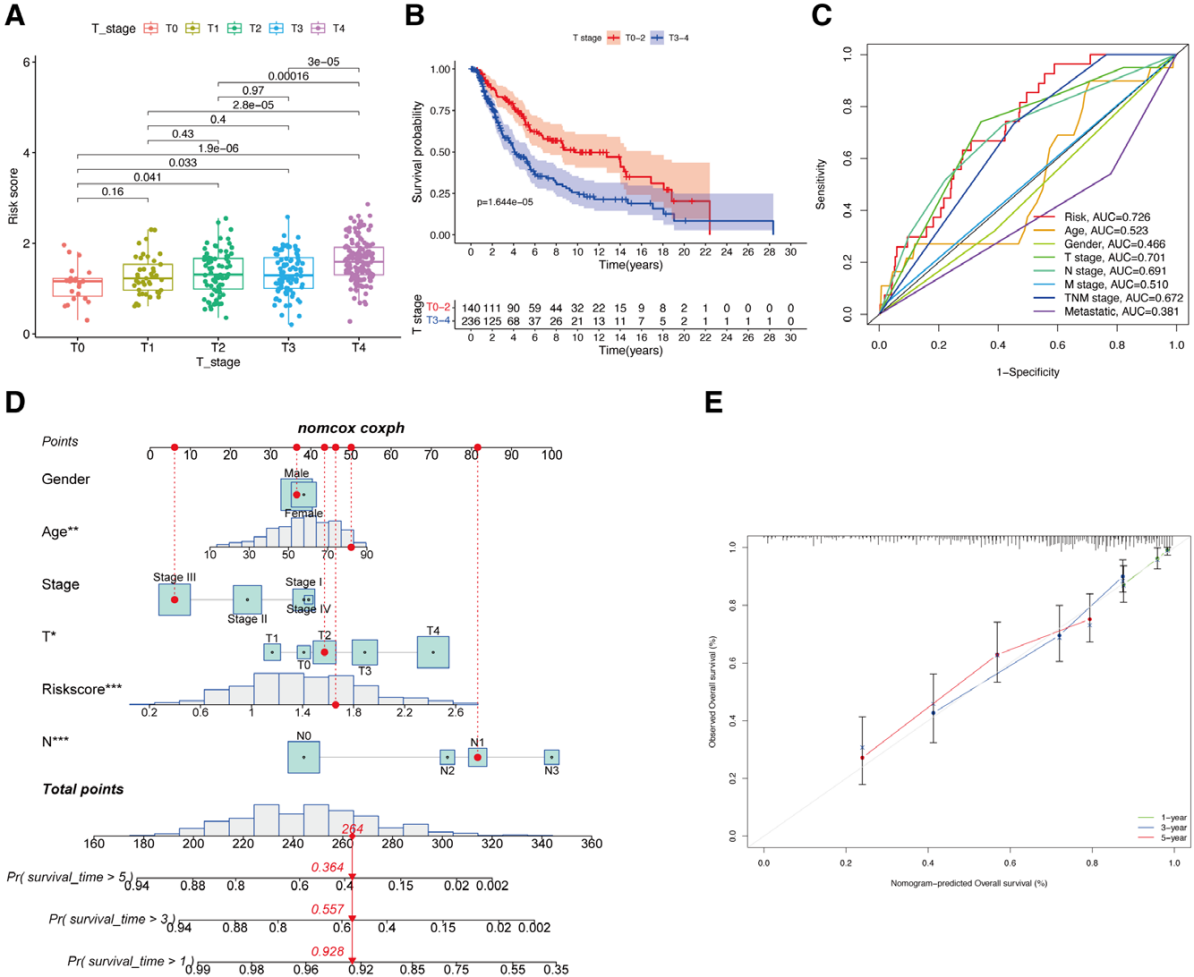
Associations between risk signature and clinicopathological factors. (A) Correlations between risk scores and T stages. (B) The prognosis of CM patients under different T stages. (C) The ROC curve of risk signature and clinical characteristics. (D) Nomogram for predicting the OS of risk scores. (E) Decision curve analysis of OS nomogram. CM = cutaneous melanoma, OS = overall survival, ROC = receiver operating characteristic.

Additionally, compared to patients with lower T stage, the patients with higher T stage exhibited obviously higher risk scores (*P* < .05; Fig. 3A). An exploration was conducted into the prognostic value of the risk signature in CM patients, encompassing various clinical characteristics. In comparison to the patient in T3-4 stage, a significant advantage of OS was observed in the CM patients with T0-2 (*P* < .05; Fig. 3B). Based on these analyses, there appears to be a correlation between the risk signature and the onset of CM. Additionally, in comparison to alternative clinical characteristics, our risk signature demonstrated stronger predictive efficacy (Fig. 3C). These results support the notion that our ECD-associated gene risk model offers a more sensitive prediction of CM patients’ OS. Finally, the nomogram plot of CM patients was created by incorporating the clinical characteristics and risk score (Fig. 3D), with the aim of utilizing the prognostic value of risk signature. Results showed that the established nomogram had exhibited substantial agreement, as demonstrated in Figure 3E.

### 3.3. Relationship of the ECD-associated gene risk signature with immune microenvironment

**Figure 4.**
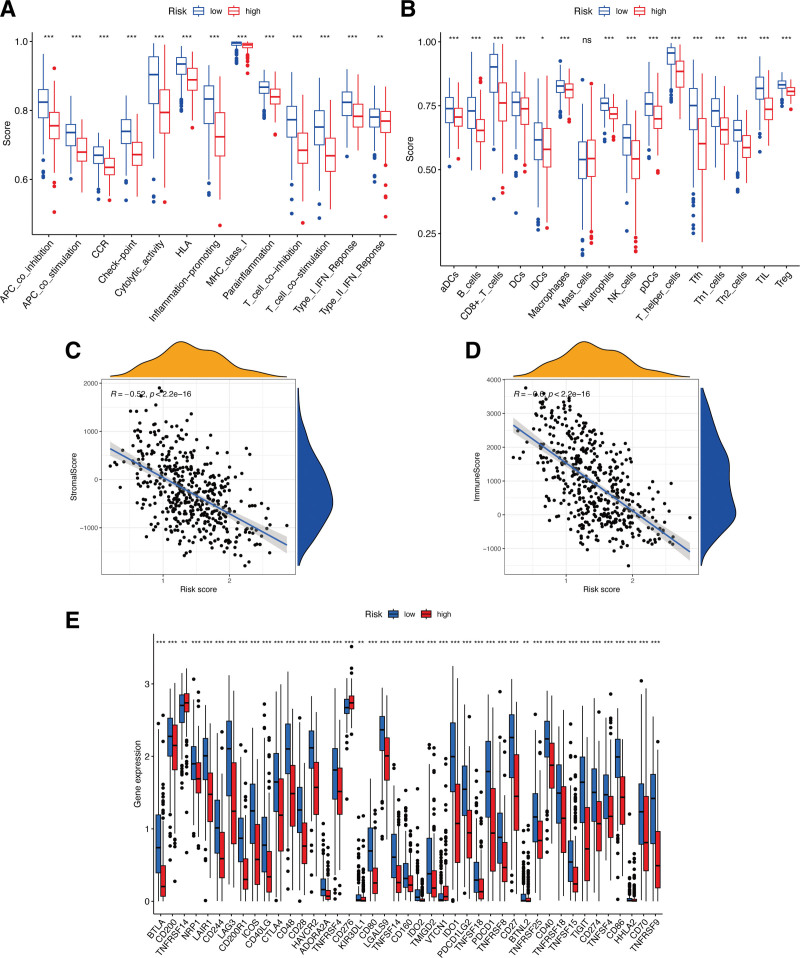
Immune characteristics of risk signature. Boxplots of scores of immune-associated functions (A) and immune cells (B) in risk signature. Associations between risk signature, stromal scores (C) and immune scores (D). (E) Expression of immune checkpoints among 2 risk subgroups in CM patients. CM = cutaneous melanoma.

In comparison to those patients with low risk scores, the patients with high risk scores exhibited a significantly reduced immune cell functions and subpopulations (*P* < .05; Fig. 4A and B). Next, the associations of the risk signature with immune microenvironment were further clarified, and the results showed a significantly negative connection between our constructed risk signature to stromal (*P* < .05; Fig. 4C) and immune (*P* < .05; Fig. 4D) scores. Within the 2 risk subgroups, immune checkpoints displayed notable differential expression, with several immune-associated genes exhibiting statistically significant differences (*P* < .05; Fig. 4E). At the same time, CM patients with high risk scores showed a marked reduction in the expression of almost all immune biomarkers, like *BTLA, CD200, NRP1*, and so on, except for *TNFRSF14* and *CD276*.

### 3.4. Construction of machine learning models and identification of hub ECD-associated gene

**Figure 5.**
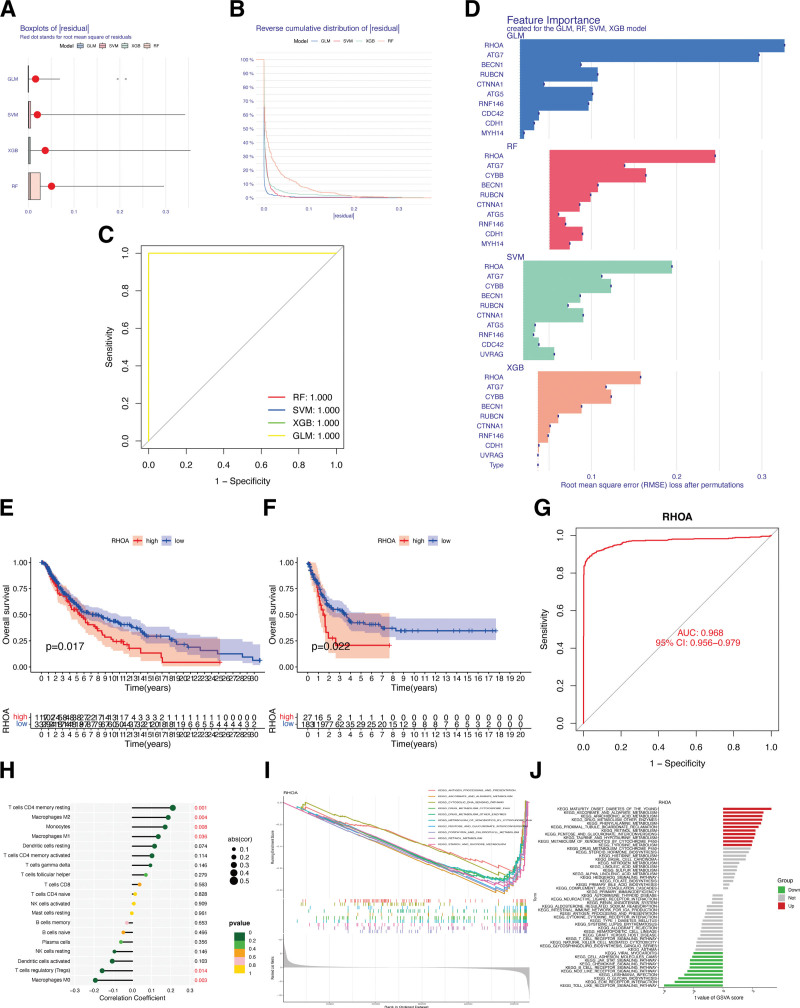
Identification of hub ECD-associated gene in CM by machine learning models. (A) Box plots of sample residuals of the 4 algorithms. (B) The reverse cumulative distribution map of model residuals. (C) ROC analysis of algorithms. (D) Importance score of feature genes in the model. The KM curve of gene *RHOA* in TCGA (E) and GSE65904 (F) CM patients. (G) ROC curve showed the prediction accuracy of *RHOA* for CM. (H) Correlations between infiltrated immune cells and *RHOA* in CM. GSEA (I) and GSVA (J) analyses of *RHOA*. CM = cutaneous melanoma, ECD = entotic cell death, ROC = receiver operating characteristic, TCGA = The Cancer Genome Atlas.

In this study, to develop diagnostic models, we employed 4 different algorithms, namely Extreme Gradient Boosting, Support Vector Machine, Random Forest tree, and Generalized Linear Models. The analyses of these models, including box plots (Fig. 5A) and reverse cumulative distribution (Fig. 5B), consistently showed small residuals between 0 and 0.1 for all 4 algorithms used. The results of ROC curves confirmed the high accuracy of the diagnostic models, as evidenced by the AUC values, which were all close to 1 for the 4 algorithms employed (Fig. 5C). In the evaluation of the importance scores of the 4 algorithms, the gene *RHOA* showed the highest score (Fig. 5D). Consequently, *RHOA* was identified as the hub ECD-associated gene in CM and was employed for subsequent prognostic analysis. To further confirm the role of *RHOA* in CM prognosis, KM analysis indicated that the expression of *RHOA* was significantly negatively connected with the overall survival (OS) of CM patients in both TCGA and GSE65904 cohorts. CM patients in the high *RHOA* expression subgroup had a significantly poorer prognosis (Fig. 5E and F). Meanwhile, the AUC values for *RHOA* were 0.968, revealing that *RHOA* could specifically predict CM occurrence (Fig. 5G). The correlation between *RHOA* and immune cells showed that the core gene *RHOA* was significantly positively connected with the infiltration of CD4 memory resting T cells, M2/M1 macrophages, and monocytes, but significantly negatively associated with regulatory T cells and M0 macrophages infiltration in CM samples (Fig. 5H). Moreover, we also observed tight associations between *RHOA* and several pathways, including antigen processing and presentation, ascorbate and aldarate metabolism, Toll-like receptor signaling pathway, ECM receptor interaction, and others, from our GSVA and GSEA enrichment analysis (Fig. 5I and J).

### 3.5. Identification of hub ECD-associated lncRNAs in CM

**Figure 6.**
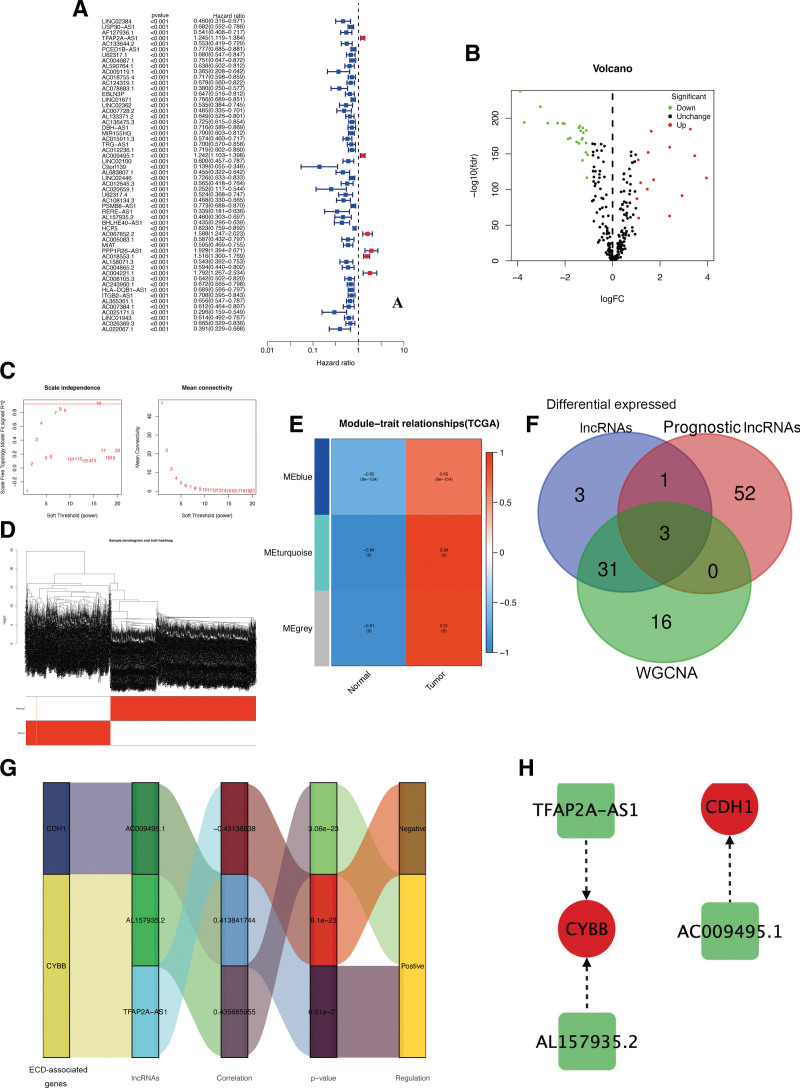
Characterization of hub ECD-associated lncRNAs in CM. (A) Univariate Cox regression analysis showed prognostic lncRNAs. (B) Volcano plot of differentially expressed lncRNAs. (C and D) Soft-thresholding powers scale-free fit index. (E) Heatmap showing the correlation between clinical traits and gene module. (F) The Venn diagram of genes among differentially expressed lncRNAs, prognostic lncRNAs, and WGCNA lists. (G–H) Correlation network of hub lncRNAs and their associated ECD-associated genes. CM = cutaneous melanoma, ECD = entotic cell death.

The researchers employed correlation analysis with |R2| > 0.4 and *P* < .05 to identify 230 lncRNAs significantly correlated with ECD genes (Table S1, Supplemental Digital Content, http://links.lww.com/MD/K573), which were then utilized for further investigations. Then, 56 prognosis-associated lncRNAs (Fig. 6A) and 38 differentially expressed lncRNAs (Fig. 6B) were identified through screening. To gain deeper insights into the functional clusters linked to CM patients, WGCNA analysis was extended to include ECD-associated lncRNAs. After considering the scale-free R2 = 0.93, the optimal soft-threshold power (β = 16, Fig. 6C) was established, resulting in the identification of 3 modules in WGCNA: blue, turquoise, and gray (Fig. 6D). The turquoise module exhibited the strongest association (*r* = 0.94) between the 2 clusters, prompting the choice of lncRNAs from this module for subsequent analysis (Fig. 6E). Based on the above analyses, 3 overlapping lncRNAs were selected as core lncRNAs for further prognostic analysis (Fig. 6F), and in Figure 6G and H, you can find the graphical representations of how the identified hub lncRNAs are connected to ECD-associated genes.

### 3.6. Role of ECD-associated lncRNAs in CM

**Figure 7.**
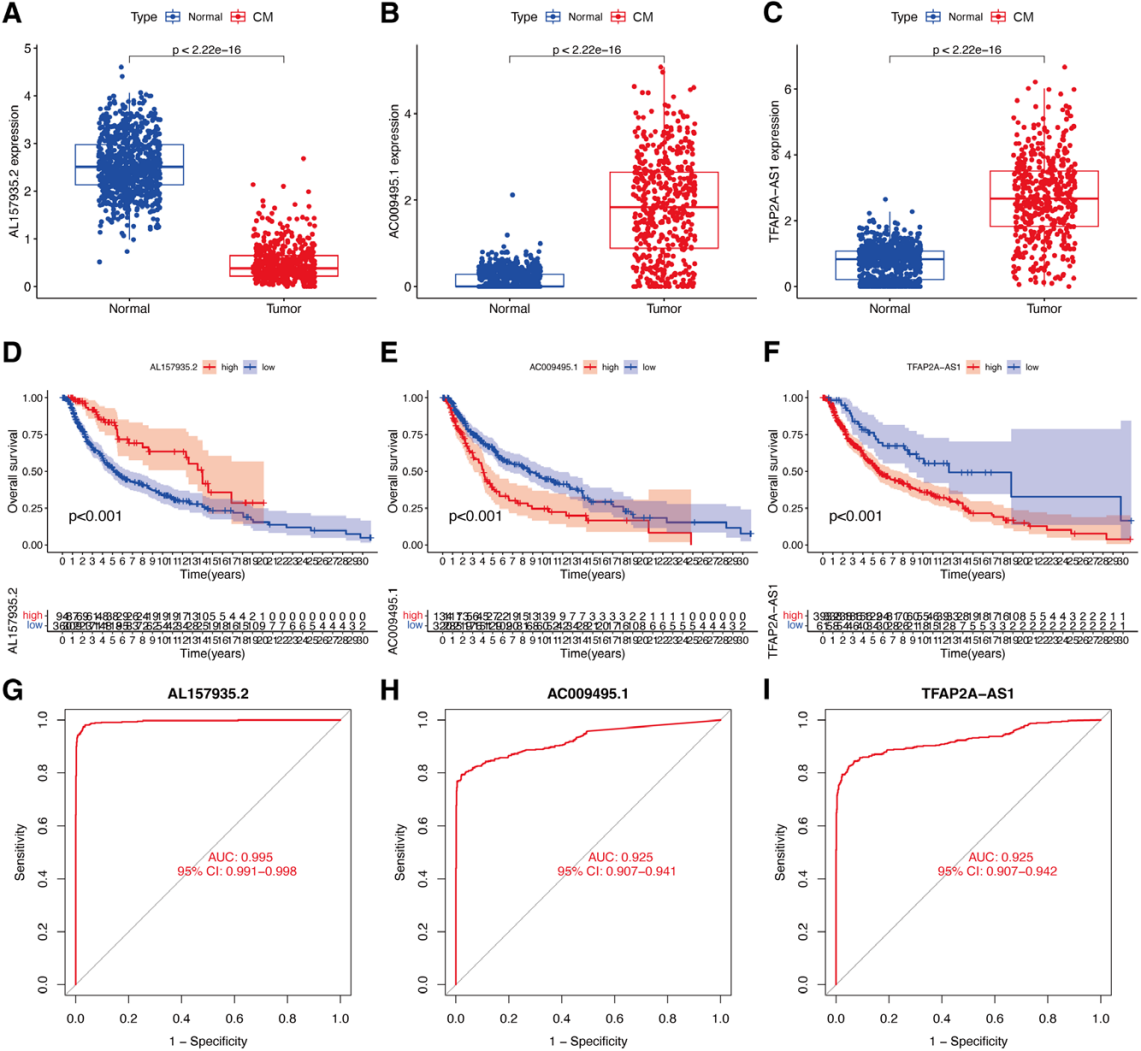
The role of ECD-associated lncRNAs in CM. (A–C) Gene expression of hub lncRNAs. (D–F) The KM curve of hub lncRNAs. (G–I) ROC curve of hub lncRNAs. CM = cutaneous melanoma, ECD = entotic cell death, ROC = receiver operating characteristic.

The results of the gene expression analysis for hub lncRNAs in CM revealed a significant downregulation of lncRNA *AL157935.2* (Fig. 7A) in CM tissues compared to normal samples (*P* < .05). Conversely, *AC009495.1* (Fig. 7B) and *TFAP2A-AS1* (Fig. 7C) were significantly upregulated in CM tissues (*P* < .05), unlike the rest of the lncRNAs. According to the KM survival analysis, CM patients with elevated *AL157935.2* expression (Fig. 7D) experienced a significantly improved overall survival (OS) (*P* < .05). On the other hand, increased expression of *AC009495.1* (Fig. 7E) and *TFAP2A-AS1* (Fig. 7F) were found to be significantly associated with an adverse prognosis in CM patients (*P* < .05). The ROC analysis indicated that, as individual diagnostic biomarkers, *AL157935.2* exhibited an AUC of 0.995 (Fig. 7G), while *AC009495.1* and *TFAP2A-AS1* displayed AUCs of 0.925 each (Fig. 7H and I). These findings highlight the high predictive accuracy of the 3 identified lncRNAs in CM patients.

### 3.7. ECD-associated lncRNAs risk signature value in CM clinics

**Figure 8.**
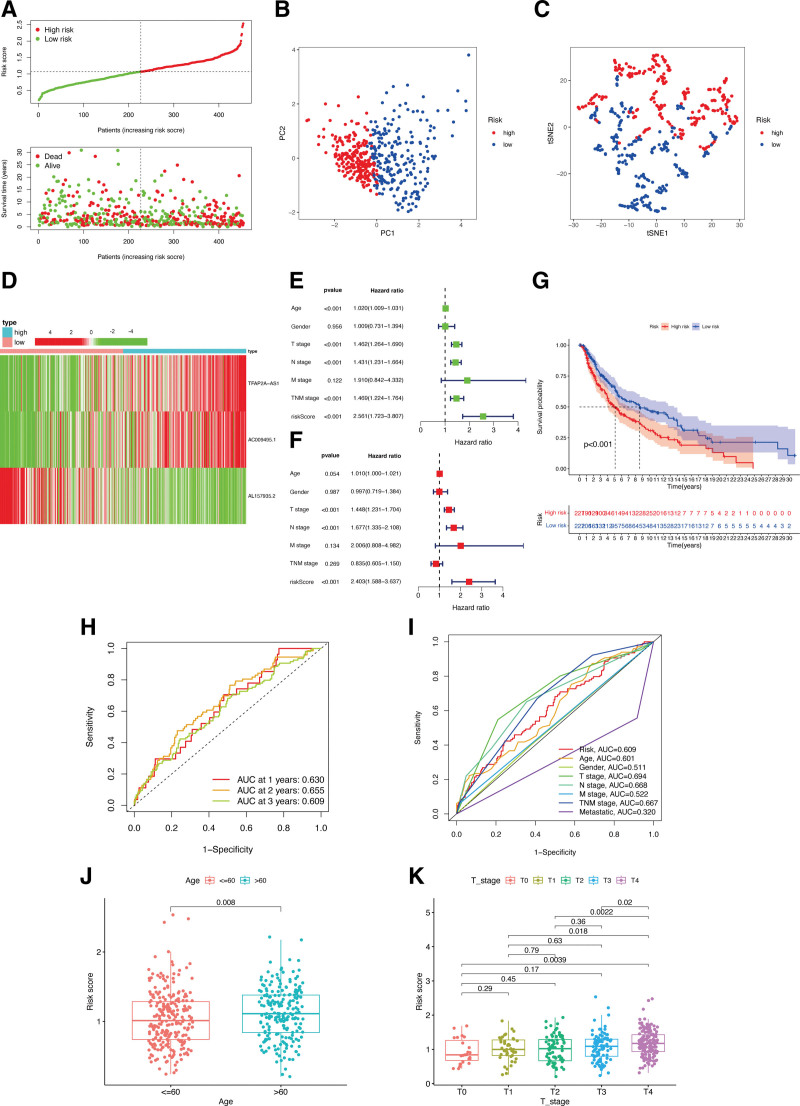
Associations between ECD-associated lncRNAs risk signature and clinicopathological factors. Risk score distribution (A), PCA plot (B), and t-SNE (C) analysis of TCGA-CM cohort. (D) The heatmap of lncRNAs expression in 2 risk subgroups. Univariate (E) and multivariate Cox (F) regression of risk signature. (G) Survival curve of CM patients. TimeROC (H) and ClinicalROC (I) curves to forecast OS of patients. Correlations between risk scores and ages (J), and T stage (K). CM = cutaneous melanoma, ECD = entotic cell death, ROC = receiver operating characteristic, TCGA = The Cancer Genome Atlas.

Based on the median value of the calculated risk scores by the identified ECD-associated lncRNAs, CM patients were classified into 2 subgroups (Fig. 8A). Clear separation of these 2 subgroups was observed in the PCA (Fig. 8B) and t-SNE (Fig. 8C) analyses. During the investigation of the connection between ECD-associated lncRNAs and the CM signature, it was observed that lncRNAs *AC009495.1* and *TFAP2A-AS1* were higher expressed in the high-risk subgroup, whereas *AL157935.2* was lower expressed in such subgroup (Fig. 8D). Furthermore, our analysis provided evidence that the risk signature of ECD-associated lncRNAs could independently predict outcomes for CM patients (Fig. 8E and F). High-risk scores in CM patients were associated with a considerably reduced OS compared to low-risk scores (*P* < .05; Fig. 8G). According to ROC analysis (Fig. 8H), the risk signature exhibited a moderate predictive accuracy at 1 (AUC = 0.63), 2 (AUC = 0.655), and 3 (AUC = 0.609) years. When contrasted with other traditional clinicopathological features (age, gender, combined or separated TNM stage, and metastatic status), the risk signature demonstrated superior accuracy in clinical ROC curve analysis (Fig. 8I), underscoring its sensitivity and specificity in predicting CM patient survival. Moreover, compared to patients with lower T stage or aged ≤ 60, the patients with higher T stage or aged > 60 exhibited obviously higher risk scores (Fig. 8J and K).

## 4. Discussion

The application of advanced sequencing technologies in the field of biology has led to the discovery of a growing number of biomarkers that are associated with melanoma. Nonetheless, there is still a pressing need to identify biomarkers with the potential for early detection or prognostic prediction among patients with CM. ECD, a newly identified mechanism for cell death, is believed to play a crucial role in cancer progression.^[11]^ On the contrary, the precise involvement of ECD in the pathogenesis and clinical behavior of CM, including its contribution to tumorigenesis, disease progression, and metastatic spread, remains largely elusive and warrants further investigation. Herein, we identified the hub ECD-associated gene *RHOA* and core ECD-associated lncRNAs in CM. Meanwhile, risk signatures were constructed based on the expression of ECD-associated genes and lncRNAs, and their high precision for predicting the OS of CM patients was further demonstrated. In addition, we observed significant correlations of ECD-associated genes and risk signatures with the immune microenvironment, indicating their potential utility and clinical advantages.

To elucidate the relationships between ECD and the OS of patients with CM, we conducted a systematic analysis of ECD-associated genes. It was discovered that all these genes showed significant differential expression in CM samples compared to normal skin. Additionally, by utilizing 4 mechanism learning algorithms and ROC analysis, *RHOA* exhibited a high importance score in predicting CM compared to other ECD-associated genes, with a high predictive accuracy and significant prognostic value for CM. Based on these findings, *RHOA* was identified as the hub ECD-associated gene in CM.

As small GTPases, the proteins of the Rho family participate in various processes, such as cellular cycle, migration, and adhesion.^[14]^ *RHOA* is one of the most canonical and extensively studied members of the Rho family. It not only functions in physiological processes but also plays a significant role in cancer prognosis and development mechanisms, including metastasis, invasion, apoptosis, and proliferation.^[15–18]^ Previous studies have highlighted the tumor-suppressive activity of *RHOA* in CM through modulating immune response and cancer cell apoptosis.^[19–21]^ However, recent functional studies have demonstrated conflicting conclusions, suggesting that pathological overexpression of *RHOA* may promote CM migration and melanoma cell survival.^[22–24]^ Thus, the specific role and signaling mechanism of *RHOA* in CM remain elusive. In this study, our results demonstrate a strong negative association between *RHOA* and the OS of patients with CM. We also observed higher *RHOA* expression in CM. This finding may be related to the modulation of immune cells and pathways. Immunotherapy has been recognized as one of the most promising therapeutic approaches for improving outcomes in various types of malignant tumors.^[25]^ In this regard, we observed enrichments of *RHOA* in immune-associated pathways, including antigen processing and presentation, B cell receptor signaling pathway, Toll-like receptor signaling pathway, and more. Additionally, the expression of *RHOA* in CM was positively correlated with the infiltration of immune cells such as CD4 memory resting T cells, M2/M1 macrophages, and monocytes infiltration, whereas it exhibited negative correlations with the infiltration of regulatory T cells and M0 macrophages. Considering the pivotal effect of immune cells on the regulation of immune response against tumors,^[26]^ this observed correlation with immune infiltration holds significant value in predicting the OS of CM patients by *RHOA*. However, the functional mechanism of *RHOA* with immune cells still needs further investigation.

Afterward, 11 ECD-associated genes (*ATG5, ATG7, BECN1, CDC42, CDH1, CTNNA1, CYBB, MYH14, RHOA, RNF146*, and *UVRAG*) and 3 ECD-associated lncRNAs (*AL157935.2, AC009495.1*, and *TFAP2A-AS1*) were utilized for constructing risk signatures, aiming to predict CM prognosis. Various methods were utilized to confirm the utility of this constructed risk signature in predicting CM prognosis. This risk signature was closely linked to CM overall survival and T stage, as observed in our study. As a clinicopathological parameter, the American Joint Committee on Cancer staging system is commonly applied in the assessment of tumors.^[27]^ In predicting CM growth and prognosis, our risk signatures showed superior accuracy compared to the TNM stage. Furthermore, a nomogram analysis confirmed the efficacy of the ECD-associated genes risk signature in predicting CM outcomes.

Furthermore, significant connections of ECD-associated genes risk signature to immune microenvironment were confirmed by several methods, the link to immune processes indicates its potential as a prognostic indicator. It is noteworthy that CM patients with high-risk scores showed marked decreases in immune cell infiltration and compromised immune functions, affecting nearly all cell types. Acknowledging the vital functions of immune cells in anti-tumor immunity,^[26]^ we speculate that the high-risk CM patients have profoundly suppressed anti-tumor immune responses. Furthermore, CM patients with high-risk scores exhibited significantly decreased expression of numerous immune checkpoint molecules, underscoring the predictive potential of the ECD-associated genes risk signature in influencing immune checkpoint expressions and its potential applicability as a reference for CM immunotherapy. Nonetheless, more investigation is warranted to examine the interactions of these genes with immune-related genes.

Although this study identified lncRNAs and hub genes associated with ECD in CM and proposed risk signatures highlighting their potent prognostic value across multiple analyses, it is important for us to acknowledge certain limitations that should be taken into consideration. Firstly, all microarray and clinical CM data used in this study were sourced from publicly-available websites, and thus, our findings need to be validated through additional experimental assays. Secondly, to validate the findings of our retrospective study, additional prospective investigations are warranted. Finally, further investigations are necessary to clarify the precise role and potential mechanisms of hub ECD-associated genes and lncRNAs in the development of CM.

In summary, this study has contributed to a better understanding of the functions of ECD in the pathogenesis of CM. The core gene *RHOA* and hub ECD-associated lncRNAs *AL157935.2, AC009495.1*, and *TFAP2A-AS1* have the potential to serve as biomarkers for the prognosis of CM patients and reflect their immune conditions. To our knowledge, this study was the first bioinformatic analysis examining the role of ECD in CM, providing a unique perspective for the advancement of therapeutic strategies for CM patients.

## Author contributions

**Data curation:** Chen Zhang.

**Formal analysis:** Chen Zhang.

**Investigation:** Chen Zhang.

**Methodology:** Chen Zhang.

**Project administration:** Chen Zhang.

**Software:** Chen Zhang.

**Writing – original draft:** Chen Zhang.

**Writing – review & editing:** Chenyang Shen.

## Supplementary Material


