# Supplementary material for: Identification of hub gene and lncRNA signature related to entotic cell death in cutaneous melanoma for prognostic and immune prediction
Source: Medicine (Baltimore). 2023 Nov 10;102(45):e35881. doi: 10.1097/MD.0000000000035881 (PMC10637533; doi:10.1097/MD.0000000000035881)
Supplement: Supplementary file 1 [file medi-102-e35881-s001.docx]

Supplementary table 1. Identification of lncRNAs correlated with ECD genes.

| **ECD genes** | **LncRNAs** | **Correlation** | **p-value** | **Regulation** |
| --- | --- | --- | --- | --- |
| CYBB | LINC02384 | 0.52326074 | 1.83E-34 | postive |
| ATG7 | AC093297.2 | -0.4372177 | 2.07E-23 | negative |
| ATG5 | AC008608.2 | -0.4000112 | 1.59E-19 | negative |
| CYBB | AC004847.1 | 0.61645002 | 1.23E-50 | postive |
| CYBB | USP30-AS1 | 0.70668254 | 1.76E-72 | postive |
| ATG7 | AL109811.2 | -0.4281086 | 2.05E-22 | negative |
| CYBB | AF127936.1 | 0.78617592 | 4.41E-100 | postive |
| CYBB | AC004921.1 | 0.59314995 | 4.29E-46 | postive |
| ATG7 | HOMER3-AS1 | 0.41858913 | 2.10E-21 | postive |
| ATG7 | AL050341.2 | -0.4243035 | 5.24E-22 | negative |
| CYBB | TFAP2A-AS1 | -0.4313664 | 9.10E-23 | negative |
| CDH1 | AC100786.1 | 0.42850784 | 1.86E-22 | postive |
| RNF146 | AL583856.2 | 0.40991917 | 1.64E-20 | postive |
| ATG5 | PIK3CD-AS2 | -0.4485116 | 1.09E-24 | negative |
| CYBB | PIK3CD-AS2 | -0.4119988 | 1.01E-20 | negative |
| ATG5 | TRAF3IP2-AS1 | 0.42819406 | 2.01E-22 | postive |
| RNF146 | TRAF3IP2-AS1 | 0.53653128 | 1.82E-36 | postive |
| ATG7 | AC010201.2 | -0.4417619 | 6.41E-24 | negative |
| ATG5 | AC006942.1 | -0.424726 | 4.73E-22 | negative |
| CDC42 | AC006942.1 | -0.408211 | 2.43E-20 | negative |
| RHOA | AC006942.1 | -0.4050856 | 5.01E-20 | negative |
| CYBB | AC104083.1 | 0.50466483 | 8.39E-32 | postive |
| CYBB | AL163051.1 | -0.4199982 | 1.49E-21 | negative |
| CYBB | AC245128.3 | 0.4543297 | 2.29E-25 | postive |
| BECN1 | AL031587.1 | 0.42230838 | 8.53E-22 | postive |
| ATG7 | AC008870.2 | -0.468218 | 4.89E-27 | negative |
| CYBB | AC133644.2 | 0.62153796 | 1.11E-51 | postive |
| ATG5 | AL139246.5 | -0.4034286 | 7.32E-20 | negative |
| CYBB | AL442663.3 | -0.4179509 | 2.45E-21 | negative |
| CYBB | AC138207.5 | 0.71371084 | 1.55E-74 | postive |
| CYBB | AC110995.1 | 0.47537281 | 6.29E-28 | postive |
| RNF146 | LINC00641 | 0.43847562 | 1.50E-23 | postive |
| CYBB | LINC01150 | 0.54363595 | 1.42E-37 | postive |
| ATG7 | AC009120.2 | -0.4076192 | 2.79E-20 | negative |
| RHOA | AC098484.1 | 0.40559222 | 4.46E-20 | postive |
| ATG7 | THAP9-AS1 | -0.4331572 | 5.80E-23 | negative |
| CYBB | MMP2-AS1 | 0.54352773 | 1.47E-37 | postive |
| RNF146 | AL356599.1 | 0.43933071 | 1.20E-23 | postive |
| CDC42 | AL117332.1 | -0.419308 | 1.76E-21 | negative |
| CYBB | PCED1B-AS1 | 0.75221238 | 5.27E-87 | postive |
| ATG7 | AL031714.1 | -0.4424392 | 5.38E-24 | negative |
| BECN1 | AC005288.1 | 0.4940865 | 2.33E-30 | postive |
| RHOA | AC005288.1 | 0.41994297 | 1.51E-21 | postive |
| ATG7 | RAD51-AS1 | -0.4378414 | 1.76E-23 | negative |
| ATG7 | GABPB1-AS1 | -0.401092 | 1.25E-19 | negative |
| ATG7 | AC084824.4 | -0.512756 | 6.11E-33 | negative |
| CDC42 | NORAD | 0.40079822 | 1.33E-19 | postive |
| ATG5 | AC138696.2 | -0.4660433 | 9.04E-27 | negative |
| CDC42 | AC138696.2 | -0.4378938 | 1.74E-23 | negative |
| RHOA | AC138696.2 | -0.4200268 | 1.48E-21 | negative |
| CYBB | U62317.1 | 0.46161511 | 3.11E-26 | postive |
| CYBB | AC004687.1 | 0.50413222 | 9.95E-32 | postive |
| ATG5 | AL133338.1 | 0.52109211 | 3.81E-34 | postive |
| RNF146 | AL133338.1 | 0.45135355 | 5.11E-25 | postive |
| CYBB | TRBV11-2 | 0.64688517 | 3.53E-57 | postive |
| ATG5 | AC092747.4 | 0.40053814 | 1.41E-19 | postive |
| ATG5 | NAPA-AS1 | -0.4013791 | 1.17E-19 | negative |
| ATG5 | POLR2J4 | -0.4178297 | 2.52E-21 | negative |
| CDC42 | POLR2J4 | -0.420617 | 1.29E-21 | negative |
| ATG7 | N4BP2L2-IT2 | -0.4428498 | 4.83E-24 | negative |
| ATG7 | AL355488.1 | -0.4138858 | 6.44E-21 | negative |
| CYBB | AL590764.1 | 0.75257238 | 3.93E-87 | postive |
| CYBB | AC009119.1 | 0.59928601 | 2.97E-47 | postive |
| CYBB | AC011472.1 | -0.4017438 | 1.07E-19 | negative |
| ATG7 | AP002954.1 | 0.42140314 | 1.06E-21 | postive |
| CYBB | AP002954.1 | 0.5910553 | 1.06E-45 | postive |
| CYBB | WDR86-AS1 | 0.41942304 | 1.72E-21 | postive |
| ATG7 | AC004908.3 | -0.4729297 | 1.27E-27 | negative |
| CYBB | AC023510.2 | 0.43435169 | 4.29E-23 | postive |
| ATG7 | ANKRD10-IT1 | -0.498306 | 6.27E-31 | negative |
| RNF146 | ANKRD10-IT1 | 0.42510108 | 4.31E-22 | postive |
| CYBB | AC097359.2 | -0.4115344 | 1.12E-20 | negative |
| ATG7 | AC009022.1 | -0.4197876 | 1.57E-21 | negative |
| ATG7 | ZKSCAN2-DT | -0.458868 | 6.64E-26 | negative |
| RNF146 | AL035530.2 | 0.40890966 | 2.07E-20 | postive |
| CYBB | AC018755.4 | 0.68864628 | 1.79E-67 | postive |
| ATG7 | ALG13-AS1 | -0.403162 | 7.78E-20 | negative |
| ATG7 | AC083843.3 | -0.4136406 | 6.82E-21 | negative |
| CDC42 | LINC01772 | 0.40843044 | 2.31E-20 | postive |
| RNF146 | AL354892.3 | 0.47719126 | 3.70E-28 | postive |
| RUBCN | MIR503HG | -0.4424421 | 5.37E-24 | negative |
| CYBB | AC124319.1 | 0.53602288 | 2.18E-36 | postive |
| CYBB | AC078883.1 | 0.4361246 | 2.73E-23 | postive |
| CYBB | AC008760.2 | 0.491626 | 4.95E-30 | postive |
| ATG5 | SNHG11 | -0.413638 | 6.83E-21 | negative |
| CDC42 | SNHG11 | -0.4141056 | 6.11E-21 | negative |
| CYBB | SNHG11 | -0.4831107 | 6.47E-29 | negative |
| CDH1 | LINC01443 | 0.4706305 | 2.46E-27 | postive |
| ATG7 | AP003352.1 | -0.4492404 | 8.98E-25 | negative |
| ATG5 | EBLN3P | 0.48111397 | 1.17E-28 | postive |
| CYBB | UBE2D3-AS1 | -0.4009583 | 1.28E-19 | negative |
| ATG7 | AC000123.1 | -0.4441069 | 3.48E-24 | negative |
| CDC42 | SLC12A9-AS1 | -0.401448 | 1.15E-19 | negative |
| CYBB | LINC01871 | 0.63879945 | 2.28E-55 | postive |
| ATG7 | AC048341.2 | -0.4773805 | 3.50E-28 | negative |
| ATG7 | AC005261.1 | -0.4047621 | 5.40E-20 | negative |
| CYBB | AC023794.4 | 0.45955984 | 5.49E-26 | postive |
| CYBB | AL158071.4 | 0.40032628 | 1.48E-19 | postive |
| CYBB | LINC02362 | 0.48767827 | 1.65E-29 | postive |
| ATG7 | AC095057.3 | -0.4284922 | 1.87E-22 | negative |
| CYBB | AC010247.1 | 0.43348546 | 5.34E-23 | postive |
| ATG7 | AC010834.3 | -0.4028422 | 8.37E-20 | negative |
| CYBB | LINC00861 | 0.60250363 | 7.14E-48 | postive |
| ATG7 | AC018638.7 | -0.4012104 | 1.21E-19 | negative |
| CYBB | AC002091.2 | 0.60895042 | 3.91E-49 | postive |
| CYBB | AC007728.2 | 0.71412257 | 1.17E-74 | postive |
| CYBB | AC009950.1 | 0.42740814 | 2.44E-22 | postive |
| RNF146 | AL355297.3 | 0.40548471 | 4.57E-20 | postive |
| CDH1 | AL034376.1 | 0.43591804 | 2.88E-23 | postive |
| CDH1 | LHFPL3-AS1 | 0.41936591 | 1.74E-21 | postive |
| ATG5 | AL451085.2 | -0.4714377 | 1.95E-27 | negative |
| ATG7 | LINC01355 | -0.4399955 | 1.01E-23 | negative |
| CYBB | AL133371.2 | 0.75315282 | 2.45E-87 | postive |
| ATG7 | STAG3L5P-PVRIG2P-PILRB | -0.4347882 | 3.84E-23 | negative |
| CYBB | AC136475.3 | 0.42969055 | 1.38E-22 | postive |
| ATG7 | AL132989.1 | -0.4804238 | 1.43E-28 | negative |
| ATG7 | PSPC1-AS2 | -0.4026537 | 8.74E-20 | negative |
| RHOA | AC022150.4 | 0.42101868 | 1.17E-21 | postive |
| CYBB | DBH-AS1 | 0.46178547 | 2.97E-26 | postive |
| CYBB | MIR155HG | 0.60769863 | 6.91E-49 | postive |
| ATG7 | AC027319.1 | 0.40366385 | 6.94E-20 | postive |
| ATG7 | AC087752.4 | -0.4232664 | 6.76E-22 | negative |
| ATG7 | AL928654.2 | -0.4537907 | 2.65E-25 | negative |
| ATG7 | AC011899.2 | 0.43568236 | 3.06E-23 | postive |
| CYBB | AC011899.2 | 0.71547602 | 4.62E-75 | postive |
| CYBB | AC015911.3 | 0.67048063 | 8.67E-63 | postive |
| CYBB | TRG-AS1 | 0.57084057 | 4.46E-42 | postive |
| CYBB | AC012236.1 | 0.50269054 | 1.57E-31 | postive |
| RHOA | AL121832.2 | -0.4014622 | 1.15E-19 | negative |
| ATG7 | OCIAD1-AS1 | -0.4076972 | 2.74E-20 | negative |
| CDH1 | AC009495.1 | 0.41384174 | 6.51E-21 | postive |
| RNF146 | AL133406.2 | 0.40070216 | 1.36E-19 | postive |
| ATG7 | LINC01004 | -0.5121003 | 7.57E-33 | negative |
| CYBB | LINC02100 | 0.44572934 | 2.27E-24 | postive |
| ATG7 | LINC00342 | -0.4630871 | 2.07E-26 | negative |
| CYBB | C9orf139 | 0.5328829 | 6.60E-36 | postive |
| ATG5 | HMGN3-AS1 | 0.40621146 | 3.87E-20 | postive |
| ATG7 | AL022328.2 | -0.4016807 | 1.09E-19 | negative |
| CYBB | AL683807.1 | 0.58666011 | 6.82E-45 | postive |
| CYBB | AL117209.1 | -0.4106862 | 1.37E-20 | negative |
| ATG7 | AL139089.1 | -0.4449803 | 2.77E-24 | negative |
| CYBB | LINC02446 | 0.6700075 | 1.14E-62 | postive |
| CYBB | AC012645.3 | 0.61508342 | 2.32E-50 | postive |
| CYBB | AC020659.1 | 0.44580309 | 2.23E-24 | postive |
| CYBB | GEMIN7-AS1 | -0.4236481 | 6.15E-22 | negative |
| ATG7 | AC002091.1 | 0.42256952 | 8.01E-22 | postive |
| CYBB | AC002091.1 | 0.59470101 | 2.20E-46 | postive |
| CYBB | AC145098.1 | 0.52342899 | 1.73E-34 | postive |
| ATG7 | NEAT1 | -0.4180518 | 2.39E-21 | negative |
| CYBB | LINC01857 | 0.62464563 | 2.50E-52 | postive |
| ATG7 | AL390728.6 | -0.5074354 | 3.45E-32 | negative |
| RNF146 | AL390728.6 | 0.40636109 | 3.74E-20 | postive |
| ATG7 | AC120053.1 | -0.4791645 | 2.08E-28 | negative |
| ATG5 | FOXN3-AS1 | -0.4030062 | 8.06E-20 | negative |
| CYBB | U62317.4 | 0.49346625 | 2.82E-30 | postive |
| ATG5 | MANEA-DT | 0.49152532 | 5.11E-30 | postive |
| RNF146 | MANEA-DT | 0.50462606 | 8.50E-32 | postive |
| ATG7 | AC092119.2 | -0.4675317 | 5.94E-27 | negative |
| CYBB | AC108134.3 | 0.62848172 | 3.89E-53 | postive |
| CYBB | PSMB8-AS1 | 0.50676633 | 4.28E-32 | postive |
| ATG7 | LINC00653 | -0.4354392 | 3.25E-23 | negative |
| ATG7 | AC127024.4 | -0.4546478 | 2.10E-25 | negative |
| ATG7 | AC093788.1 | -0.4503334 | 6.71E-25 | negative |
| CYBB | RERE-AS1 | 0.41736331 | 2.81E-21 | postive |
| ATG7 | AC008735.2 | -0.4902157 | 7.62E-30 | negative |
| CYBB | AL157935.2 | 0.43568596 | 3.06E-23 | postive |
| CYBB | PXN-AS1 | -0.4561105 | 1.41E-25 | negative |
| CYBB | BHLHE40-AS1 | 0.68737615 | 3.91E-67 | postive |
| CYBB | AC064836.3 | -0.4058601 | 4.19E-20 | negative |
| ATG5 | AC021078.1 | 0.40655655 | 3.57E-20 | postive |
| ATG7 | AC021078.1 | -0.4738292 | 9.83E-28 | negative |
| RNF146 | Z97989.1 | 0.5286279 | 2.91E-35 | postive |
| CYBB | HCP5 | 0.59547903 | 1.57E-46 | postive |
| ATG7 | MIR4453HG | -0.4084233 | 2.32E-20 | negative |
| CTNNA1 | AC092354.2 | 0.40880229 | 2.12E-20 | postive |
| RNF146 | AL049552.1 | 0.43885808 | 1.36E-23 | postive |
| ATG5 | AC067852.2 | -0.472063 | 1.63E-27 | negative |
| CDC42 | AC067852.2 | -0.4137984 | 6.57E-21 | negative |
| CYBB | AC005083.1 | 0.59747932 | 6.55E-47 | postive |
| ATG7 | PSMA3-AS1 | -0.480638 | 1.35E-28 | negative |
| CYBB | AC007611.1 | -0.4197538 | 1.58E-21 | negative |
| ATG5 | PCAT6 | -0.4011177 | 1.24E-19 | negative |
| CYBB | PCAT6 | -0.4300321 | 1.27E-22 | negative |
| CYBB | AL359076.1 | 0.43078024 | 1.05E-22 | postive |
| CYBB | MIAT | 0.62080303 | 1.57E-51 | postive |
| CYBB | PPP1R26-AS1 | -0.4440365 | 3.54E-24 | negative |
| CYBB | AC018553.1 | -0.4339806 | 4.71E-23 | negative |
| CYBB | AL158071.3 | 0.45689553 | 1.14E-25 | postive |
| CYBB | AC003070.1 | 0.53345772 | 5.39E-36 | postive |
| ATG5 | YTHDF3-AS1 | -0.4389435 | 1.33E-23 | negative |
| CYBB | YTHDF3-AS1 | -0.4281102 | 2.05E-22 | negative |
| ATG5 | AL359715.2 | 0.4320404 | 7.68E-23 | postive |
| RNF146 | AL359715.2 | 0.46581721 | 9.63E-27 | postive |
| ATG7 | SNHG1 | -0.4098946 | 1.65E-20 | negative |
| ATG7 | AC007066.2 | -0.4445382 | 3.11E-24 | negative |
| ATG5 | AL359715.1 | 0.44498077 | 2.77E-24 | postive |
| RNF146 | AL359715.1 | 0.50030193 | 3.35E-31 | postive |
| ATG7 | AC114730.3 | -0.4120496 | 9.93E-21 | negative |
| ATG7 | AC002553.1 | -0.4320412 | 7.68E-23 | negative |
| CYBB | AC004865.2 | 0.59649234 | 1.01E-46 | postive |
| ATG5 | FGD5-AS1 | 0.48124119 | 1.13E-28 | postive |
| CDC42 | FGD5-AS1 | 0.47923241 | 2.04E-28 | postive |
| RHOA | FGD5-AS1 | 0.55327421 | 4.01E-39 | postive |
| ATG5 | ZNF687-AS1 | -0.4084413 | 2.31E-20 | negative |
| ATG7 | MALAT1 | -0.4118374 | 1.04E-20 | negative |
| ATG7 | AC127024.5 | -0.4045315 | 5.69E-20 | negative |
| CYBB | AC093278.2 | 0.4076658 | 2.76E-20 | postive |
| CYBB | AC004221.1 | -0.4234222 | 6.50E-22 | negative |
| ATG7 | AC008972.2 | 0.40485009 | 5.29E-20 | postive |
| ATG5 | AC040169.1 | -0.4185036 | 2.14E-21 | negative |
| ATG7 | UBR5-AS1 | -0.4121414 | 9.72E-21 | negative |
| CYBB | AC008105.3 | 0.53247111 | 7.62E-36 | postive |
| RNF146 | AL390208.1 | 0.49417266 | 2.26E-30 | postive |
| CYBB | AC243960.1 | 0.66312143 | 5.53E-61 | postive |
| CYBB | AL161785.1 | 0.58884114 | 2.71E-45 | postive |
| ATG7 | SENCR | 0.4551392 | 1.84E-25 | postive |
| CYBB | SENCR | 0.41147704 | 1.14E-20 | postive |
| ATG7 | Z83843.1 | -0.4940197 | 2.37E-30 | negative |
| RNF146 | Z83843.1 | 0.40524749 | 4.83E-20 | postive |
| CDC42 | SUCLA2-AS1 | -0.4134074 | 7.21E-21 | negative |
| CYBB | TSPOAP1-AS1 | 0.42752835 | 2.37E-22 | postive |
| CDC42 | AC011451.1 | 0.41385958 | 6.48E-21 | postive |
| CYBB | C1RL-AS1 | 0.45136925 | 5.08E-25 | postive |
| CYBB | AC068473.5 | -0.4189177 | 1.94E-21 | negative |
| ATG7 | AC004253.1 | -0.4075506 | 2.84E-20 | negative |
| ATG5 | LINC01547 | -0.4272202 | 2.56E-22 | negative |
| ATG5 | AC133552.5 | -0.400502 | 1.42E-19 | negative |
| CYBB | LINC01094 | 0.71967432 | 2.50E-76 | postive |
| CYBB | HLA-DQB1-AS1 | 0.60931745 | 3.31E-49 | postive |
| CDH1 | AC110285.1 | 0.43759327 | 1.88E-23 | postive |
| CYBB | AC017002.3 | 0.43719481 | 2.08E-23 | postive |
| CYBB | ITGB2-AS1 | 0.6447023 | 1.10E-56 | postive |
| CYBB | AC090559.1 | 0.81826544 | 7.59E-115 | postive |
| CYBB | AL365361.1 | 0.69715507 | 8.66E-70 | postive |
| ATG7 | SNHG4 | -0.4216097 | 1.01E-21 | negative |
| ATG7 | AC110285.2 | -0.4019271 | 1.03E-19 | negative |
| ATG5 | NOP14-AS1 | -0.4460057 | 2.11E-24 | negative |
| CYBB | AC015819.1 | 0.55362667 | 3.51E-39 | postive |
| CYBB | CARD8-AS1 | 0.47437481 | 8.39E-28 | postive |
| CYBB | AC007384.1 | 0.63619417 | 8.50E-55 | postive |
| ATG7 | AC068620.2 | -0.467352 | 6.25E-27 | negative |
| CYBB | AL135818.1 | 0.62971807 | 2.12E-53 | postive |
| CYBB | AC022706.1 | 0.60791271 | 6.27E-49 | postive |
| CYBB | AC025171.5 | 0.44199746 | 6.03E-24 | postive |
| CYBB | LINC01943 | 0.63034627 | 1.56E-53 | postive |
| CYBB | AC004585.1 | 0.49761241 | 7.79E-31 | postive |
| ATG5 | AP003068.1 | -0.4548431 | 1.99E-25 | negative |
| CDC42 | AP003068.1 | -0.4274708 | 2.40E-22 | negative |
| CYBB | AC127521.1 | 0.60675354 | 1.06E-48 | postive |
| CYBB | HSD11B1-AS1 | 0.43219052 | 7.40E-23 | postive |
| ATG7 | AC002553.2 | -0.4721884 | 1.58E-27 | negative |
| CYBB | AC026369.3 | 0.57703899 | 3.67E-43 | postive |
| ATG7 | AC005332.3 | -0.4032098 | 7.70E-20 | negative |
| CYBB | AL109741.1 | 0.40979504 | 1.68E-20 | postive |
| CYBB | AL022067.1 | 0.47132827 | 2.02E-27 | postive |
| ATG7 | ZNF32-AS1 | -0.413552 | 6.97E-21 | negative |
| CYBB | AL139020.1 | 0.44959616 | 8.17E-25 | postive |
| ATG7 | AC132192.2 | -0.4038476 | 6.65E-20 | negative |
